# Emotional Function, Negative Thoughts about the Pandemic, and Adaptability Skills among Dementia Caregivers during the COVID-19 Pandemic

**DOI:** 10.3390/brainsci12040459

**Published:** 2022-03-29

**Authors:** Evdokia Nikolaidou, Marianna Tsatali, Marina Eleftheriou, Helen Wang, Konstantina Karagiozi, Petrina Margaritidou, Magdalini Tsolaki

**Affiliations:** 1 Greek Association of Alzheimer Disease and Related Disorders (GAADRD), K. Karamanli 164, 54248 Thessaloniki, Greece; nikolaidou.e@alzheimer-hellas.gr (E.N.); marina.eleftheriou@gmail.com (M.E.); dinakaragiozi@gmail.com (K.K.); pmargaritidou7@yahoo.gr (P.M.); tsolakim1@gmail.com (M.T.); 2 Laboratory of Neurodegenerative Diseases, Center for Interdisciplinary Research and Innovation, Aristotle University of Thessaloniki (CIRI-AUTh), 54124 Thessaloniki, Greece; 3 Department of Psychology, School of Social Sciences, University of Ioannina, 45110 Ioannina, Greece; 4 Psychology Department, University of Princeton, Princeton, NJ 08540, USA; hw10@princeton.edu; 5 1st Department of Neurology, Faculty of Health Sciences, School of Medicine, Aristotle University of Thessaloniki, 54124 Thessaloniki, Greece

**Keywords:** adaptability skills, Coronavirus-19 pandemic, dementia caregivers, emotional management

## Abstract

**Background**: It can be easily inferred that dementia caregivers were severely affected by the general home confinement, or ‘lockdown’, due to their caregiving roles. **Aim**: The aim of the current study is to investigate how the quarantine measures applied during the COVID-19 pandemic affected the emotional function (specifically the emotional management, emotional experience, and stress symptoms) and the negative thoughts, as well as the adaptability skills, of Greek dementia caregivers. **Materials andMethods**: In total, 303 participants (138 in the non-caregiving adults-control group; 165 in the dementia caregivers-experimental group) were recruited from the day centers of the Greek Association of Alzheimer Disease and Related Disorders (GAADRD) from June 2020 to January 2021 in Thessaloniki, Greece. The caregiving population was split into group 1 (those who participated in support groups) and group 2 (those who did not participate in support groups). A self-reported questionnaire was created for research purposes and was digitally administered to participants via Google forms. **Results**: During the quarantine period, dementia caregivers had more difficulties in emotional management, especially in regards to stress symptoms, which was measured via the Beck Anxiety Inventory (BAI), in comparison to the control group. In regards to the caregiving populations, caregivers from group 1 were more able to manage their emotions according to their self-reports, but had increased agony and loneliness. Moreover, statistical significance was found between emotional management, negative feelings, and stress symptoms in those from group 2 who self-reported that the PwD deteriorated. This evidence was not found in group 1. Finally, there were no differences in the means of negative thoughts about the pandemic, as well as in the adaptation skills, both in dementia caregivers and in the control group, according to their self-reports. **Discussion**: It seems that different emotional aspects were affected in the dementia caregivers’ population, and, therefore, future psychotherapeutic interventions should focus on those most severely affected by the restrictive measures imposed.

## 1. Introduction

Declared a public health threat by the World Health Organization (WHO) on 11 March 2020 [1], the 2019 Coronavirus (COVID-19) outbreak led to unprecedented worldwide closures as the disease swept across multiple continents [2]. Various governments implemented lockdown procedures and encouraged (and, in many cases, mandated) citizens to employ various safety protocols in attempts to curb the disease’s growth and lower both infection and mortality rates [2]. The overall nature of the pandemic has led to increased social isolation, loneliness, and inactivity, while also compromising people’s ability to access food, resources, and social support. In many cases, the pandemic has also directly impacted employment, financial security, and general access to medical services [1]. Consequently, the impact of the disease outbreak can be felt by many, if not all, populations [3].

### 1.1. Mental Health Crisis

COVID-19 has been found to be detrimental for mental health. Most longitudinal studies [1] have found evidence of increased depression, anxiety, and stress following the pandemic. Sanchez-Gomez et al. [4] also noted signs of hyperarousal and intrusive thoughts, as well as symptoms of post-traumatic stress, noting that hypervigilance appeared to be the most prevalent response to the pandemic rather than avoidance.

Early evidence from the pandemic suggests that some groups are more heavily impacted by the pandemic than others. O’Connor et al. [5], in their study, surveyed 3077 adults in the United Kingdom over the course of the first six weeks of lockdown. They found increased levels of suicidal ideation and depressive symptomology over time but also noted that participants reported decreased anxiety. Feelings of defeat and entrapment also decreased, while ratings for positive well-being increased. They, however, also identified specific subgroups that appeared to experience worse mental health outcomes than most, with the data indicating that women, young people, socially disadvantaged groups, and individuals with pre-existing mental health conditions were more likely to feel the impacts of the pandemic more greatly.

Frontline health workers have been especially increasingly reporting declines in mental health over the course of the pandemic. Martin et al. [6] looked at 2089 health care workers in Spain in a cohort study. Of the participants, 38.58% met criteria for clinical depression, 51.75% for clinical anxiety, 60.4% for clinical stress, and 21.57% for clinical insomnia. Martin et al. [6] concluded that interaction with COVID-19 was the main explanatory variable for the rise and noted that the worst outcomes were amongst health care workers that identified as nursing home workers. Finally, Sanchez-Gomez et al. [7] (2021) state that health professionals with a higher self-perception of emotional intelligence were more engaged in their working duties, which had a positive impact on their overall work performance. Therefore, their study highlights the necessity of promoting self-efficacy in health-care workforce due to high emotional demands in this workplace. In line with the aforementioned findings, Moreno et al. (2020) [1] highlight the significance of improving mental health services due to the increased challenges raised by the COVID-19 pandemic.

### 1.2. Patients with Dementia

Caregivers of patient(s) with dementia (PwD) have been placed in an especially difficult position by the pandemic. Due to the general demographic category (older adults) occupied by the PwD, as well as the physiological vulnerabilities that are often comorbid with it, PwDs are more vulnerable to COVID-19 than most. Some studies have shown that pre-existing dementia was the highest risk factor for severe COVID-19 symptomology in patients over 65 years old [8]. The ApoE ε4 genotype, a genotype predictive of dementia and specifically Alzheimer’s disease, was also linked to more severe reactions to COVID-19 infection [9].

Beyond physiological vulnerabilities [10], PwDs often have impaired abilities to abide by current COVID-19 safety protocols, such as wearing masks, following social distancing recommendations, practicing hand hygiene, and monitoring COVID-19 symptomology. This increases their likelihood of infection, if not cared for properly by their caregivers.

Decreased access to health care services following the pandemic has also compromised their care. Inability to receive proper testing, neuroimaging, and therapeutics has, in many cases, postponed diagnosis and compromised treatment. Social distancing measures have also been reported to even worsen the effects of dementia in some cases. PwDs have been reported to have increased cognitive decline, often exhibiting increased agitation, depression, apathy, and anxiety. Moreover, it is worth mentioning that, according to the longitudinal study of Salfi et al. (2021) [11], living with a high-risk person for COVID-19 infection was a risk category for poor sleep quality and worse mental health. This is also supporting by other studies, which pointed to a different time course of sleep and mental health between genders during the home confinement period, specifically, women seemed to show greater long-term resilience during the lockdown (Salfi et al., 2020) [12].

### 1.3. Caregivers before and after the Pandemic

Even outside of the extenuating circumstances of the pandemic, research has shown that caregivers of PwD typically have poor mental health. Many caregivers suffer from compassion fatigue and often exhibit signs of stress, anxiety, and depression [13,14]. This has only worsened over the pandemic. The pandemic-induced quarantine often meant extended contact with the PwD, which allowed for more meaningful interactions between caregiver and patient but also meant higher rates of burnout for caregivers [9]. Salva et al. [15], for instance, conducted a study on 123 caregivers of PwDs in southwest Virginia and found that some reported that their PwDs were more at ease since their caregivers were at home more often. The same study, however, also discovered that 41% of caregivers felt weary due to the reduction in assistance they had.

In an online survey of 94 Italian caregivers of PwDs, Altieri & Santangelo [16] report observing increasing severe depressive symptomology over the course of the pandemic. The same trend was not observed with anxiety, but 50% of caregivers reported that their lifestyles were impacted by the COVID-19 pandemic. Interestingly, enough, 73.8% of caregivers also reported that their patients were unaware of the pandemic.

Budnick et al. [17] conducted a similar study with German caregivers, where they examined the impact of the coronavirus pandemic on informal caregivers. They recruited 1000 informal caregivers between 40 and 85 years old. In general, most caregivers reported little to no changes in their care situation. However, caregivers of PwDs and those who typically relied on professional assistance were disproportionally more likely to report increased burdens following the arrival of the pandemic. They typically were more negative, had more concerns and demands, and were more likely to report a loss of support. Such caregivers were also more likely to report having issues implementing COVID-19 safety measures.

Carpinelli et al. [18] looked at the effect the COVID-19 lockdown had on the psychological outcome of caregivers, while also examining the effect of the loss of welfare services. They looked at over 239 Italian participants. Of those, 43% reported a loss or discontinuation of support and/or assistance, with 42% losing professional nurses and domestic aids. In general, caregivers were increasingly depressed and/or anxious over the lockdown, though Carpinelli et al. [18] observed that education level served to safeguard against it.

### 1.4. Coping Strategies

The literature has established that caregivers of PwDs have, overall, suffered from increased care burden as a consequence of the pandemic, with many of them displaying classic signs of depression. Alves et al. [19] conducted a review assessing various measures that could be used to alleviate this. They reviewed 43 studies that utilized various psychoeducational and psychosocial interventions. They found that interventions were helpful when they addressed restlessness, apathy, and other behavioral symptoms. In the context of the current pandemic, the authors promoted monitoring sleep, daily walks, and light exposure to counter the side effects of quarantine. It was noted that telephone-based interventions were particularly effective as they provided caregivers with regular support and assistance. Similar results were shown with friend–technology devices.

Other surveys on caregivers indicated that outside of psychological interventions, they were actively taking measures to cope with their stress. Some methods were directed at addressing the mood and treatment of the PwD in question. Caregivers mentioned using methods such as increasing medication dosages, doing physical activity, seeing pictures, walking outside, making videos, and taking time to meditate/breathe with their charges [20]. Other methods were more self-directed. In specific, Salva et al. [15] noted that 57% of the caregivers they studied used active coping strategies such as taking personal time, going outside alone, gardening, and making masks for care aides. Another 43% of caregivers used more passive methods, such as spending increased time on their cellphones, playing games, etc.

Based on the aforementioned literature, we expected the following:

**Hypothesis** **1a.** *Compared to the control group (people who are not caregivers), dementia caregivers will be more likely to report more stress symptoms and negative thoughts about the pandemic during the COVID-19 lockdowns. They will also be more likely to have poorer emotional management and adaptability skills*.

**Hypothesis** **1b.** *Caregivers who participated in support groups will differ from those who did not attend such groups in regard to the aforementioned variables.*

**Hypothesis** **1c.** *Caregivers will report that the quarantine has affected their care of PwDs.*

## 2. Methods

### 2.1. Participants

The study’s participants were recruited from various regions of Greece—urban and/or non-urban areas—as well as from Greek populations abroad. Specifically, from the total sample of 303 participants, 165 were dementia caregivers [experimental group; 76 participated in supportive groups delivered digitally from the GAADRD from March 2020 to June 2021 (group 1), whereas 89 did not participate in these groups (group 2)], and 138 were aged adults (control group). Participants’ recruitment was randomized. It is noteworthy that both groups were recruited between September 2021 and October 2021.

Participants from group 1 were recruited from the Family Unit of the Day Care Center “Saint Helen” as well as the Home Care Unit of the Greek Association of Alzheimer Disease and Related Disorders (GAADRD). This unit is the section of the GAADRD that provides education and support to caregivers, helping them cope with the diagnosis of dementia. The unit teaches caregivers how to manage their own emotional and physical burden, in addition to the behavioral disturbances common for PwDs. It is worth mentioning that during the COVID-19 pandemic, as well as because of the restrictive measures imposed, participants from group 1 continued to participate in the support groups which were provided through web platforms, more specifically Skype, Viber, and Zoom. Regarding group 2, they were recruited from the head psychologist of the GAADRD, as caregivers typically accompanied the PwD to be tested. After the PwD’s final evaluation, their caregivers were asked about whether they would like to participate in support groups, and if they would like to take part in the forthcoming study. No caregivers were denied access to the support groups. Participants in group 2 were typically assigned as such because they indicated that they lacked the availability to attend the support group. All participants were primary caregivers of PwDs, irrespective of etiology, since the participants’ type of dementia was not considered in the selection of our sample. PwDs had been initially diagnosed with dementia in moderate stage by the certified neurologist of the GAADRD. Participants from the control groups were recruited through the announcements being published by the authors of the current study through social media. It is noteworthy that none of the participants who belonged to the control group provided any kind of care to another person, due to dementia or any other chronic illness. More details about the sample’s demographics and characteristics are shown in Table 1, Table 2 and Table 3.

### 2.2. Material

The self-reported questionnaire used in this study, was created by the psychologists of the Caregivers’ Department of the Greek Alzheimer Association and Related Disorders (GAADRD) for research purposes. The questions referred to the dependent variables of the study (*emotional function*, emotional management, emotional experience, stress symptoms, and *negative thoughts about the pandemic*, as well as *adaptability skills* and, finally, the *psychosocial effect of the quarantine on PwDs, according to caregivers’ self-reports*) were placed in dual response answers (‘yes’–‘no’), e.g., emotional experience (agony, sadness, fear, anxiety, frustration, calmness, loneliness, guilt, activation, optimism, hope, sense of protection, burden, other), as well as 5-point Likert scales, e.g., measuring emotional management (‘very difficult’, ‘difficult’, ‘neutral’, ‘easy’, ‘very easy’), and negative thoughts (‘not at all’, ‘little’, ‘enough’, ‘much’, ‘very much’). The Beck Anxiety Inventory (BAI) [21] was administered in order to evaluate stress symptoms. Finally, the skills variable was divided into (i) the ability to deal with various circumstances during the lockdown period as well as the restrictive measures imposed (‘very difficult’, ‘difficult’, ‘neutral’, ‘easy’, ‘very easy’) and (ii) the ability to adapt to difficult situations during the pandemic (‘very difficult’, ‘difficult’, ‘neutral’, ‘easy’, ‘very easy’). The psychosocial effect of the quarantine on PwDs, according to caregivers’ self-reports, was split into the following variables: observed changes in PwDs, ease of adapting to health care measures, ease of which PwDs understood the pandemic as well as the lockdown measures, and the possible medication change in PwDs across the two groups of our sample (for more details, please see the Appendix A Questionnaire).

In order to test our hypothesis, we created two different self-reported questionnaires administered separately to the control group as well as the experimental group. Both questionnaires had the same questions, however, the one administered to the dementia caregivers included questions relating to their relationship with the PwD and the possible effects of the quarantine on PwD care (see Appendix A). 

The questionnaire was digitally administered via Google forms due to the restrictive measures imposed during the COVID-19 pandemic. The questionnaires were delivered via the GAADRD profile on Facebook as well as the official email account, to those who had previously contacted the GAADRD and signed that they would be willing to take part in research endeavors. Before the questionnaire’s administration, participants read the information sheet, including the research purpose, and signed the informed consent about their voluntary participation in the study, as well as the data protection and their anonymity. The questionnaires’ completion lasted for 10 min.

The study was approved by the Scientific and Ethics Committee of the GAADRD (Scientific Committee Approved Meeting Number: 62/12-12-2020), which follows the new General Data Protection Regulation (EU) 2016/679 of the European Parliament and of the Council of 27 April 2016 on the protection of natural persons, with regard to the processing of personal data and on the free movement of such data, as well as the principles outlined in the Helsinki Declaration.

### 2.3. Statistical Analysis

The statistical analysis was initially performed using SPSS software version 27 (IBM; SPSS Statistics for Windows, Version 27.0. Armonk, NY, USA: IBM Corp 27.0). Initially, descriptive statistics (means and SDs) were calculated in order to describe the levels of dependent variables (*emotional function during the pandemic*; emotional management, emotional experience, stress symptoms; *negative thoughts* and *adaptability skills*; ease of dealing with difficult situations, and ease of adapting to difficult situations during the pandemic, as well as *the psychosocial effect of the quarantine on PwDs according to caregivers’ self-reports*). The Independent Samples *T*-test was applied to identify whether the groups differed by means of age and education years, as well as their BAI total scores. Additionally, the chi-square test of independence was performed on quantitative, dichotomous, and categorical variables to examine the relation between the groups of our study, by means of our aforementioned dependent variables. The level of significance was set at *p* < 0.05. However, for multiple comparisons, such as in the analysis of emotional experience, the level of significance was set at *p* < 0.05.

## 3. Results

### 3.1. Descriptive Statistics

In detail, the experimental group (n = 165) consisted of two different sub groups: (a) (group 1) 76 caregivers that participated in support groups (14 men and 56 women, age range: 37 to 49 years, *M* = 45.12, *SD* = 4.25, education range: 6 to 20 years [primary education (1.3%); secondary education (36.8%); University graduates (35.5%); Master graduates (25%); Doctorate graduates (1.3%)]) and (b) (group 2) 89 caregivers who did not attend virtual supporting groups (14 men and 75 women, age range: 35 to 48 years, *M* = 46.49, *SD* = 5.51, education range: 6 to 20 years [primary education (1.0%); secondary education (38.2%); University graduates (38.2%); Master graduates (20.2%); Doctorate graduates (2.0%)]). The control group consisted of 138 adults (21 men and 117 women, age range: 32 to 48 years, M = 46.57, SD = 7.29, education range: 12 to 20 years [secondary education (22.6%); University graduates (46.3%); Master graduates (26.8%); Doctorate graduates (4.3%)]) who did not undertake the role of caregiver during the quarantine.

The chi-square test of independence showed no significant differences between the control and experimental groups by means of gender [χ(1) = 1.020, *p* = 0.312]. Additionally, the Independent Samples T-test showed no statistically significant differences between the two groups in regards to age *t*(163) = 1.459, *p* = 0.147. However, the two groups differed by education level χ(5) = 11.701, *p* = 0.033 according to the chi-square test of independence. Therefore, the control group was more educated compared to the caregivers.

Comparing the two caregiver groups in regards to demographic variables, no differences were found for gender [χ(1) = 0.596, *p* = 0.448], age [χ(6) = 9.401, *p* = 0.150], as well as education [χ(5) = 2.605, *p* = 0.742]. Additionally, group 1 did not differ from group 2 by means of the percentage of cohabitation with the PwD [χ(1) = 0.000, *p* = 0.991], the percentage of cohabitation with the PwD during the quarantine [χ(1) = 0.621, *p* = 0.427], and the levels of sharing care of the PwD with other caregivers [χ(1) = 0.480, *p* = 0.467]. In both groups, half of the participants were the main caregivers of the PwD [χ(1) = 0.621, *p* = 0.419] (Table 1, Table 2 and Table 3). 

Differences between experimental and control groups by means of emotional function during the COVID-19 pandemic. M and SD of variables of the questionnaire are given in Table 4, Table 5 and Table 6.

### 3.2. Emotional Management

Participants were asked about how easy it was to manage their emotions during the quarantine as well as because of the confinement measures imposed. Caregivers were significantly more likely to report that it was more difficult to manage their emotions during the quarantine than the control group [χ(4) = 16.488, *p* = 0.002].

Additionally, caregivers who had psychosocial support during the quarantine (group 1) had fewer difficulties managing their emotions compared to those who did not receive similar support (group 2) [χ(4) = 9.866, *p* = 0.049] (see Table 4).

### 3.3. Emotional Experience

Caregivers had reduced levels of sadness [χ(1) = 10.492, *p* = 0.001], frustration [χ(1) = 9.718, *p* = 0.002], loneliness [χ(1) = 16.587, *p* < 0.001], and hope [χ(1) = 6.551, *p* = 0.013] when compared to the control group. Interestingly, no differences were observed in the sense of burden, which was initially expected to be increased in the caregivers’ group.

Caregivers from group 1 had increased levels of agony [χ(1) = 6.627, *p* = 0.010] and loneliness [χ(1) = 5.117, *p* = 0.031] (Table 5).

### 3.4. Stress Symptoms

Stress symptoms measured by the BAI sum score increased in the experimental groups in comparison to the controls *t*(301) = 3.204, *p* = 0.001. Moreover, caregivers from group 1 had similar levels of stress symptoms compared to those from group 2 *t*(163) = −1.191, *p* = 0.235.

### 3.5. Negative Thoughts

The levels of negative thoughts did not differ between the control and experimental groups, nor between the caregivers from groups 1 & 2 (Table 7).

### 3.6. Adaptability Skills

According to the results, caregivers did not differ from the control group in regards to the self-reported ease of dealing with difficult situations, as well as the ease of adapting to difficult situations during the pandemic and the lockdown measures implemented. Moving to the two caregiver subgroups, no differences in their adaptability skills were observed (Table 8).

### 3.7. Effect of the Quarantine in PwD Care According to Dementia Caregivers’ Self-Reports

Caregivers from both groups gave similar reports about observed changes in PwD during the pandemic (almost half of them reported that PwD deteriorated during the lockdown measures) and percentages of PwD compliance with hygiene measures, as well as ease in understanding the lockdown measures, even though the majority of them, from both groups, provided PwDs all necessary information about the pandemic. Finally, no medication changes were observed in the large majority of PwDs, according to caregivers’ self-reports from both groups.

It is worth mentioning that the variables mentioned above (Table 9) did not impact the emotional function, negative thoughts, and adaptability skills of dementia caregivers who mentioned that the PwD did not deteriorate during the pandemic. Those who mentioned that the PwD deteriorated during the pandemic were less able to manage their emotions [χ(4) = 17.141, *p* = 0.002],had more fear [χ(1) = 3.878, *p* = 0.048], and reduced optimism [χ(1) = 3.953, *p* = 0.047]. Finally, in regard to stress symptoms, they experienced increased stress levels, as reported by the BAI total score *t*(89) = 4.210, *p* < 0.001.

## 4. Discussion

In the current study, we aimed to identify whether dementia caregivers were affected by means of emotional management and emotional experience, as well as stress symptoms’ prevalence (referred as emotional function), during the COVID-19 pandemic, compared to a control group consisting of non-caregivers. Additionally, the study also attempted to investigate whether dementia caregivers had significantly increased negative thoughts and reduced adaptation skills compared to non-caregiver adults. The second goal of our study was to investigate whether dementia caregivers who participated in support groups during the pandemic, specifically March 2020–June 2021, differed by means of emotional function as well as negative thoughts and adaptability skills prevalence, in comparison to those who did not attend support groups. Finally, the extent to which the COVID-19 pandemic affected PwDs, according to caregivers’ self-reports, was the final goal of the current study.

Our results showed that during the COVID-19 pandemic, caregivers had increased difficulties in managing their emotions compared to non-caregiving populations, which is in accordance with previous studies in the literature [22,23] that stress the caregivers’ burden and subsequent emotional dysregulation. In specific, Hanna et al. [23] highlighted the long-term effects of the COVID-19 public health measures on dementia caregivers’ mental health, which should be set as a top priority in dementia care. Moreover, the fact that dementia caregivers who attended support groups during the COVID-19 pandemic were more able to manage their emotions provides significant evidence about the need to support caregivers in such circumstances, which pose unique and significant challenges for community-dwelling caregivers and PwDs. In fact, health care policies should continue implementing support groups in dementia caregivers who cannot attend onsite groups, in parallel with PwD therapeutic practices, due to the emotional burden found in this population.

Contrary to what was expected, caregivers had reduced levels of sadness, frustration, loneliness, and hope during the COVID-19 pandemic period, compared to the non-caregiver population. Therefore, it seems that being compromised with the aforementioned negative emotions in their personal lives before the current situation led them to be less affected than the general population. One possible explanation could be age, since previous studies (Viselli et al., 2021; Amicucci et al., 2021) [24,25] found that young people seem to be more at risk for mental health problems compared to older adults; however, in our study caregivers and non-caregivers were matched for age and, therefore, age cannot be attributed as a possible explanation. Hence, up to now, to our knowledge, there are no previous studies supporting this evidence, therefore, future endeavors should shed light on the above findings. Interestingly, caregivers who did not receive psychosocial support had lower levels of agony and loneliness. According to recent studies [26], accessing support groups attenuates the risk of depression and loneliness in dementia caregivers. Therefore, a possible explanation about this evidence could be that those from group 2 did not search for support from our Caregivers’ Department, since they did not have increased negative emotions, such as agony and loneliness, during the pandemic. Additionally, another reason could be that, according to our findings, those who mentioned that the PwD deteriorated during the pandemic reported that they were less able to manage their emotions and had more fear, as well as reduced optimism and increased stress symptoms, as measured by the BAI test. Hence, it can be possibly assumed that the reason of increased self-reported emotional experience in group 1 (those who participated in support groups), could be the fact that the PwD was deteriorated due to the COVID-19 pandemic.

Moving to the third aspect of the emotional function, stress symptoms, it seems that during the quarantine period, caregivers in total had increased stress levels compared to the non-caregiving population as reported in the BAI sum score. To explain the discrepancy between increased stress symptoms and reduced negative emotions observed in dementia caregivers, it is worth mentioning that stress symptoms could be partially attributed to somatic disorders, in which many of those symptoms co-exist irrespectively from anxiety levels. Hence, given that in general, dementia caregivers have increased burden, compared to the general population, increasing stress symptoms could also reflect burden issues, which could be assumed to be worsened during the pandemic. This is in line with the fact that no differences in regards to stress symptoms were observed between group 1 and group 2, which were both dementia caregivers. Another explanation could be aligned with the fact that different emotional sub-functions were affected in different ways by the pandemic, at least according to the participants’ self-reports. In fact, it can be assumed that stress symptoms are possibly related to other variables such as burden, whereas self-reported emotional experience is less affected by the pandemic, since dementia caregivers possibly face confinement more times in their daily routine. Therefore, future research should shed light on the real impact of quarantine on the caregivers’ emotional subcategories.

Moving to the rest of the variables in our study, it seems that the levels of negative thoughts as well as the levels of adaptability did not differ between caregivers, participating in support groups or not, and the control group. Hence, it could be attributed that the quarantine measures affected a vast majority of the population, irrespective of being dementia caregivers or not. Regarding previous findings, Losada et al. [27] found that the pandemic negatively affected caregivers’ perceptions about the progress of their personal negative emotions as well as negative thoughts about giving up caregiving. Finally, our results stress that negative thoughts are not limited to dementia caregivers but are also widespread in the general population obviously for different reasons. Concerning adaptability, there are few studies measuring this variable in general population; for that reason, no extensive conclusions can be extracted. According to the study by Demetriou [28], self-reported adaptability was related to increased hope and a sense of resilience, whereas adaptability was positively associated with participants’ older age and education level. However, their data sample included a general population of 18 years or older, whereas there were no comparisons with older adults and/or dementia caregivers, which would be comparable to our study. To conclude, negative thoughts and mainly adaptability measures could be studied in more detail to detect those dementia caregivers in higher need of support.

Finally, according to the results, emotional function and negative thoughts, as well as adaptability skills, were not associated with whether the PwDs’ situation was deteriorated or not. On the contrary, those from group 2 who mentioned that the PwD was deteriorated had lower ability to manage their emotions, had increased negative feelings, e.g., fear, as well as reduced optimism and stress symptoms. Therefore, those who did not participate in support groups were at greater risk of emotional distress in case their patient deteriorated, which underlines the importance of caregiving support. To our knowledge, no previous studies have been conducted to shed light on this issue.

The psychosocial impact of the COVID-19 pandemic has been broad and very challenging. The results of the current study highlight that future support groups should be mainly focused on lack of emotional management and increased stress symptoms when supporting dementia caregivers in lockdown measures or any other social restrictions, since these emotional sub-categories was found to be increased in this population as compared to the non-caregiving populations. Additionally, further research should focus on the study of negative thoughts and relevant self-reported adaptability skills, in order to investigate whether positive thoughts as well as a sense of resilience differ between the dementia population and non-caregivers, in terms of confinement situations such as those of the COVID-19 pandemic.

## 5. Conclusions

According to the findings of the present study, dementia caregivers had lower levels of emotional management as well as increased stress symptoms as compared to the non-caregiving population. Dementia caregivers who attended support groups (group 1) had increased self-reported emotional management, but also increased negative emotional experience, compared to those who did not participate in such groups (group 2). This could possibly reflect those different aspects of emotional subcategories are affected in different ways during confinement measures in dementia caregivers, irrespective of whether they participated in support groups or not. Additionally, those caregivers from group 2 who mentioned that the PwD deteriorated due to the COVID-19 pandemic had deteriorated emotional experience as well as stress symptoms, and, therefore, this could possibly explain the fact that those from group 2 self-reported lower negative emotions during the lockdown measures. Furthermore, it could be assumed that other factors such as sleep, which is particularly important for its impact on mental health, as well as for its strong influence on emotional function and regulation (Tempesta et al., 2018) [29], may influence emotional function and, therefore, provide some further explanations about the aforementioned results. However, despite the fact that sleep problems in caregivers could be a very important factor to discuss, sleep was not evaluated in the current study.

### Limitations

A possible limitation could be the representativeness of our sample. In fact, participants of the current study were not matched to education level, and, therefore, this variable could affect our results. Another limit of this study could be the absence of comparing dementia caregivers with various other caregiving population and identifying any differences in their emotional function and negative thoughts, as well as adaptability skills. Moreover, the reliability of remotely delivered and self-rated assessment measures still remains questionable, as compared to onsite evaluations. Despite that, in our previous study (Karagiozi et al., 2021) [30] we did not find any differences between onsite and online evaluation, so future endeavors should shed more light on these remote self-rated assessments. Moreover, most of the psychometric scales used were developed ad hoc for the study’s scopes, so are not assumed as valid tools. However, our sample was recruited during the first quarantine imposed, and, therefore, we aimed at identifying participants’ personal experience given by their self-report measures. Additionally, to our knowledge, there are not specific psychometric tools measuring dementia caregivers’ response to the COVID-19 pandemic due to the novelty of this situation. Furthermore, the scope of our study was to figure out the needs of the dementia caregiving population and help them in clinical practice, so this is the reason why we chose self-reports instead of other valid tools. Future studies will provide valid psychometric tools based on the subjective experience of caregivers. Finally, further research trials about any possible differences between those who had participated in support groups prior to the pandemic with those who started during the pandemic would certainly provide fruitful knowledge in this field.

## Figures and Tables

**Table 1:** Demographics in the control and experimental groups.

Demographics	Control Group (n = 138)	Experimental Group (n = 165) Participation in Supportive Groups	
		Yes (n = 76)	No (n = 89)
Gender			
Men	21(15.2%)	14 (18.4%)	14 (14.1%)
Women	117(84.8%)	56 (81.6%)	75 (85.9%)
Age (years)	45.03(6.38)	45.12 (4.25)	46.49 (5.51)
Education (level %)			
Primary education	----	1 (1.3%)	1 (1.4%)
Secondary education	31(22.6%)	28 (36.8%)	34 (38.2%)
University graduates	64(46.3%)	27 (35.5%)	34 (38.2%)
Master graduates	37(26.8%)	19 (25%)	18 (20.2%)
Doctorate graduates	6(4.3%)	1 (1.3%)	2 (2.0%)

**Table 2:** Samples’ characteristics.

Demographics	Control Group (n = 138)	Experimental Group (n = 165) Participation in Supportive Groups	
		Yes	No
Marital status			
Married	94 (68.1%)	42 (55.3%)	56 (62.9%)
Single	13 (9.3%)	23 (30.3%)	18 (20.2%)
Divorced	10 (7.6%)	7 (9.2%)	10 (11.5%)
Widow	3 (2.1%)	3 (3.9%)	4 (4.4%)
In relationship	18 (12.9%)	1 (1.3%)	1 (1.0%)
Residence			
Big city	116 (84%)	70 (92.1%)	74 (83.1%)
Province	13 (9.6%)	2 (2.6%)	10 (11.3%)
Island	3 (2.1%)	3 (3.9%)	5 (5.6%)
Abroad	6 (4.3%)	1 (1.3%)	-
Professional status			
Civil servant	29 (20.7%)	16 (21.1%)	16 (17.9%)
Private employee	46 (33.3%)	14 (18.4%)	20 (22.4%)
Freelancer	17 (12.1%)	8 (10.5%)	20 (22.4%)
Unemployed	20 (14.3%)	16 (21.0%)	18 (20.6%)
University student	5 (3.6%)		3 (3.3%)
Retired	18 (12.9%)	18 (23.7%)	10 (11.2%)
Other	3 (2.1%)	4 (5.3%)	2 (2.2%)

**Table 3:** Caregivers’ characteristics.

Demographics	Experimental Group (n = 165) Participation in Supportive Groups	
	Yes	No
Relationship with PwD		
Parent	61 (80.3%)	65 (73%)
Spouse	8 (10.5%)	5 (5.9%)
Brother/Sister	1 (1.3%)	4 (4.0%)
Relative	2 (2.6%)	1 (1.0%)
Professional caregivers	2 (2.6%)	2 (2.0%)
Grandfather/mother	-	3 (3.0%)
Parent-in-law	-	7 (7.1%)
Other	2 (2.6%)	2 (2.0%)
Living with PwD		
Yes	30 (39.5%)	39 (43.9%)
No	46 (60.5%)	50 (56.1%)
Living with PwD during pandemic		
Yes	43 (56.6%)	45 (50.6%)
No	33 (43.4%)	44 (49.4%)
Main caregiver		
Yes	43 (56.6%)	45 (50.6%)
No	33 (43.4%)	44 (56.1%)
Sharing the caregiving duties		
Yes	50 (65.8%)	60 (67.4%)
No	26 (34.2%)	29 (32.6%)

**Table 4:** Emotional function during the pandemic—*Emotional management*.

Psychosocial Effect	Control Group	Experimental Group	*p* Values	Participation in Supportive Groups		*p* Values
				Yes	No	
**Emotional management**			0.002			0.049
*Very difficult*	5 (3.6%)	27 (15.4%)		42 (55.3%)	58 (65.1%)	
*Difficult*	23 (16.9%)	40 (22.9%)		23 (30.3%)	20 (22.4%)	
*Neutral*	64 (46.3%)	55 (31.4%)		7 (9.2%)	8 (8.9%)	
*Easy*	34 (24.6%)	42 (24.0%)		3 (3.9%)	2 (2.6%)	
*Very easy*	12 (8.6%)	11 (6.3%)		1 (1.3%)	1 (1.0%)	

**Table 5:** Emotional function during the pandemic—*Emotional experience*.

Emotions	Control Group	Experimental Group	*p* Values	Participation in Supportive Groups		*p* Values
				Yes	No	
Agony			0.958			**0.010**
Yes	50 (36.3%)	60 (36.4%)		35 (46.1%)	25 (28.1%)	
No	88 (63.7%)	105 (63.6%)		41 (53.9%)	64 (71.9%)	
Sadness			**0.001**			0.694
Yes	44 (31.8%)	25 (15.2%)		14 (18.4%)	16 (18%)	
No	94 (68.2%)	140 (84.8%)		62 (81.6%)	73 (82%)	
Fear			0.528			0.635
Yes	46 (33.3%)	52 (31.5%)		25 (32.9%)	29 (32.6%)	
No	92 (66.7%)	113 (68.5%)		51 (67.1%)	60 (67.4%)	
Anxiety			0.592			0.489
Yes	61 (44.3%)	69 (41.8%)		28 (36.8%)	43 (48.3%)	
No	77 (55.7%)	96 (58.2%)		48 (63.2%)	46 (51.7%)	
Frustration			**0.002**			0.928
Yes	47 (34.1%)	30 (18.2%)		15 (19.7%)	17 (19.2%)	
No	91 (65.9%)	135 (81.8%)		61 (80.3%)	72 (80.8%)	
Calmness			0.051			0.579
Yes	24 (17.3%)	18 (11%)		9 (9.1%)	9 (10.2%)	
No	114 (82.7%)	147 (89%)		67 (90.9%)	80 (89.8%)	
Loneliness			**<0.001**			**0.031**
Yes	45 (32.7%)	23 (13.9%)		15 (19.7%)	8 (8.9%)	
No	93 (67.3%)	142 (86.1%)		61 (80.3%)	81 (91.1%)	
Guilt			0.114			0.749
Yes	2 (1.8%)	8 (4.9%)		3 (3.9%)	5 (5.1%)	
No	136 (98.5%)	157 (95.1%)		73 (96.1%)	84 (94.3%)	
Activation			0.152			0.052
Yes	13 (7.5%)	9 (5.5%)		7 (9.2%)	2 (1.9%)	
No	125 (92.5%)	156 (94.5%)		69 (90.8%)	87 (98.1%)	
Optimism			0.108			0.178
Yes	22 (15.9%)	17 (10.4%)		10 (13.2%)	7 (7.1%)	
No	116 (84.1%)	148 (89.6%)		66 (86.8%)	82 (92.9%)	
Hope			**0.013**			0.680
Yes	32 (23.2%)	21 (12.8%)		10 (13.2%)	11 (12.4%)	
No	106 (76.8%)	144 (87.2%)		66 (86.8%)	78 (87.6%)	
Sense of protection			0.556			0.125
Yes	36 (25.7%)	40 (24.3%)		21 (27.6%)	19 (21.4%)	
No	102 (73.9%)	125 (75.7%)		55 (72.4%)	70 (78.6%)	
Burden			0.133			0.052
Yes	95 (68.8%)	107 (64.9%)		47 (61.8%)	60 (67.5%)	
No	43 (31.2%)	58 (35.1%)		29 (38.2%)	29 (32.5%)	
Other			0.454			0.950
Yes	16 (11.4%)	25 (15.2%)		11 (14.5%)	14 (15.8%)	
No	122 (88.4%)	140 (84.8%)		65 (85.5%)	75 (84.2%)	

**Table 6:** Emotional function during the pandemic—*Stress Symptoms*.

Emotions	Control Group	Experimental Group	*p* Values	Participation in Supportive Groups		*p* Values
				Yes	No	
BAI_total (Μ/SD)	26.04 (8.57)	29.61 (10.23)	**0.001**	28.72 (10.26)	39.90 (12.65)	0.235

**Table 7:** Negative thoughts about the pandemic.

Psychosocial Effect	Control Group	Experimental Group	*p* Values	Participation in Supportive Groups		*p* Values
				Yes	No	
Negative thoughts			0.458			0.219
Not at all	18 (13.9%)	21 (12.7%)		8 (10.5%)	13 (14.6%)	
Little	36 (26.1%)	43 (26.06)		26 (34.2%)	20 (22.4%)	
Enough	46 (32.9%)	56 (33.9%)		23 (30.3%)	30 (33.7%)	
Much	31 (22.1%)	28 (16.9%)		14 (18.4%)	14 (15.9%)	
Very much	7 (5.0%)	17 (10.3%)		5 (6.6%)	12 (13.4%)	

**Table 8:** Adaptability skills affecting participants during the pandemic.

Demographics	Control Group	Experimental Group	*p* Values	Experimental Group Participation in Supportive Groups		*p* Values
				Yes	No	
Deal with various circumstances during the lockdown			0.585			0.431
Very difficult	4 (2.9%)	5 (3.1%)		3 (3.9%)	2 (2.2%)	
Difficult	11 (7.9%)	14 (8.4%)		4 (5.3%)	6 (6.7%)	
Neutral	77 (55.7%)	78 (47.4%)		37 (48.7%)	40 (44.9%)	
Easy	40 (28.9%)	59 (35.7%)		30 (39.5%)	34 (38.4%)	
Very easy	6 (4.6%)	9 (5.4%)		2 (2.6%)	7 (7.8%)	
Adapt in difficult situations during the lockdown			0.625			0.219
Very difficult	4 (2.8%)	7 (4.2%)		3 (3.9%)	4 (4.4%)	
Difficult	13 (9.4%)	19 (11.6%)		7 (9.2%)	6 (6.7%)	
Neutral	59 (42.9%)	68 (41.2%)		31 (40.8%)	38 (42.8%)	
Easy	55 (39.8%)	56 (33.9%)		32 (42.1%)	29 (32.7%)	
Very easy	7 (5.1%)	15 (9.1%)		3 (3.9%)	12 (13.4%)	

**Table 9:** Psychosocial effect of the quarantine in PwD—caregivers’ self-reports.

Psychosocial Effect	Participation in Supportive Groups		*p* Values
	Yes	No	
Change			0.084
Improvement	2 (2.6%)	-	
Deterioration	46 (60.5%)	45 (50.5%)	
Stable	28 (36.8%)	44 (49.5%)	
Changed domains			0.932
Cognitive	18 (23.7%)	20 (22.6%)	
Behavioral	15 (19.7%)	16 (17.9%)	
Emotional	20 (26.3%)	22 (24.7%)	
No change observed	23 (30.3%)	31 (34.8%)	
Compliance with hygiene measures			0.130
Very easy	9 (11.8%)	15 (16.8%)	
Easy	16 (21.1%)	10 (11.2%)	
Neutral	12 (15.8%)	16 (17.9%)	
Difficult	23 (30.3%)	23 (25.8%)	
Very difficult	16 (21.1%)	25 (28.3%)	
Explanation to PwD about the pandemic			0.697
Yes	63 (82.9%)	69 (77.8%)	
No	13 (17.1%)	20 (22.2%)	
Ease to understand the lockdown measures			0.538
Very easy	7 (9.2%)	11 (12.3%)	
Easy	13 (17.1%)	9 (10.3%)	
Neutral	23 (30.3%)	20 (22.4%)	
Difficult	11 (14.5%)	13 (14.6%)	
Very difficult	22 (28.9%)	36 (40.4%)	
Medication changed			0.706
Yes	14 (18.4%)	20 (22.2%)	
No	62 (81.6%)	69 (78.8%)	

## Data Availability

All data generated or analysed during this study are included in thisarticle. There are no separate or additional files.

## Appendix A. Questionnaire

### Psychosocial Effect of COVID-19 Pandemic in Dementia Caregivers

The questions placed above aim to gain further information about the PwD as well as how they experienced the COVID-19 pandemic and the lockdown measures imposed, according to the dementia caregivers’ perspective.

1. In which dementia stage does the PwD belong?
  ▪ Mild
  ▪ Moderate
  ▪ Severe
  ▪ Improved
2. Did the PwD worsen during the COVID-19 pandemic and the lockdown measures imposed?
  ▪ Improved
  ▪ Deteriorated
  ▪ Stable
3. If yes, in which domain?
  ▪ Cognitive (e.g., memory, attention, language, orientation)
  ▪ Behavioral (e.g., irritability, illusions, apathy, aggressiveness, psychomotor agitation/retardation, etc.)
  ▪ Emotional (e.g., anxiety and depressive symptoms)
4. How easy was it to comply with the proposed hygiene measures?
  ▪ Very easy
  ▪ Easy
  ▪ Neutral
  ▪ Difficult
  ▪ Very difficult
5. Did you explain the situation about the pandemic to the PwD?
  ▪ Yes
  ▪ No
6. Was it easy to understand the whole situation about the pandemic?
  ▪ Very easy
  ▪ Easy
  ▪ Neutral
  ▪ Difficult
  ▪ Very easy
  ▪ Very difficult
7. During the pandemic and lockdown measures imposed, did the PwD change medication? (any kind)
  ▪ Yes
  ▪ No
